# Sex differences in adolescent physical aggression: Evidence from sixty‐three low‐and middle‐income countries

**DOI:** 10.1002/ab.21799

**Published:** 2018-10-03

**Authors:** Amy Nivette, Alex Sutherland, Manuel Eisner, Joseph Murray

**Affiliations:** ^1^ Department of Sociology Utrecht University Utrecht The Netherlands; ^2^ RAND Europe Westbrook Centre Cambridge United Kingdom; ^3^ Institute of Criminology University of Cambridge Cambridge United Kingdom; ^4^ Postgraduate Programme in Epidemiology Federal University of Pelotas Pelotas RS Brazil; ^5^ Department of Psychiatry University of Cambridge, Forvie Site Cambridge United Kingdom

**Keywords:** physical aggression, sex differences, sexual selection, social roles

## Abstract

A great deal of research shows that adolescent and adult males are more likely to engage in physical aggression and violence than females are. However, few studies have examined cross‐cultural variation in sex differences, particularly among low‐ and middle‐income countries [LMICs]. Based on social role and sexual selection theories, we derived two hypotheses regarding possible variations in sex differences across societal contexts: 1) sex differences increase with societal gender polarization (social role theory) and 2) sex differences are exacerbated in societies where socio‐economic opportunities are scarce, unequal, or insecure (prediction derived from sexual selection theory). The current study examined the prevalence of and variation in sex differences in physical aggression, as measured by frequent fighting, among 247,909 adolescents in 63 low‐ and middle‐income countries. The results show that, overall, males were over twice as likely (OR = 2.68; 95% CI = 2.60–2.76) to report frequent fighting in the past 12 months than females. However, sex differences vary significantly across LMICs, wherein countries with higher female prevalence rates have smaller sex differences in frequent fighting. Contrary to expectations derived from social role theory, sex differences in physical aggression decrease as societal gender inequality increased. In regards to sexual selection theory, we find no evidence that sex differences in frequent fighting varies according to societal rule of law or income inequality.

## 1 INTRODUCTION

Existing evidence suggests that males are consistently more likely to engage in direct physical aggression than females (Archer, 2004, 2009; Fagan & Lindsey, 2014). In a comprehensive review of research syntheses on sex differences in direct physical aggression, covering a wide range of ages and measurement types, Archer (2004: 254) found that standardized differences in physical aggression range in magnitude from *d *= 0.33 to *d *= 0.91.

There are two dominant and competing explanations for this difference. The first explanation, known as social role theory, posits that sex differences in physical aggression are the result of socialization into gender roles that differentially prescribe the use of aggression and violence among males and females (Eagly, 1997; Eagly, Wood, & Diekman, 2000). In essence, social role theorists argue that men are more likely to be physically aggressive due to societal emphasis on stereotypical dominant and competitive roles (Archer, 2009; Eagly et al., 2000). By contrast, women are socialized into more compliant and gentle roles that discourage violence. Later versions of the theory incorporated physical sex differences as constraints on roles (Wood & Eagly, 2010, 2012). Wood and Eagly (2010, 2012) argue that evolved physical differences between males and females can account for the emergence of the stereotypical division of labor and consequently the social roles that affect the likelihood of physical aggression.

The second explanation is derived from evolutionary models of sexual selection, wherein males have greater competition for reproductive success due to lower parental investment than females (Archer, 2009; Trivers, 1972). As such, males have evolved a range of physical characteristics and psychological mechanisms that facilitate fighting over access to mates and resources (Buss & Duntley, 2006; Geary, Byrd‐Craven, Hoard, Vigil, & Numtee, 2003). Among adolescent males, physical aggression is used to achieve social dominance and effectively compete for status and resources, such as access to relationships, number of allies (peer network), and reputation or popularity (Hoff, Reese‐Weber, Schneider, & Stagg, 2009; Pellegrini, 2008).

Indeed, there is support for the notion that direct aggression is an important tool for achieving social status, popularity, and leadership among adolescent males (see e.g., Hoff et al., 2009). Females have long internal gestation periods and higher parental investment, consequently increasing the costs for direct physical aggression (Campbell, 1999; Trivers, 1972). Thus, according to sexual selection theories, the sex difference in physical aggression can be accounted for by differential evolutionary pressures rooted in differences in parental investment leading to reproductive success.

While both social role and sexual selection theories predict that males are more likely to engage in physical aggression against same‐sex within‐group competitors than females, they generate different expectations regarding the variability of sex differences across environments. From a social role perspective, sex differences are the result of social or internalized expectancies and socialization, and therefore size of the sex difference in physical aggression should vary according to the polarization of gender roles (Archer, 2009; Nivette, Eisner, Malti, & Ribeaud, 2014; Wood & Eagly, 2010, 2012). In other words, in societies with more pronounced gender roles and expectations, males are more likely to conform to the dominant, competitive stereotype, leading to more physical aggression than stereotypical compliant and gentle characteristics of females.

By contrast, sexual selection theory proposes that the magnitude of sex differences in physical aggression varies with the extent to which aggression is an effective strategy for men to gain access to resources that are important for reproductive success (Archer, 2009; see also Geary et al., 2003; Schmitt & Rohde, 2013). In addition, male physical competition is more likely to occur in societies that lack effective institutional structures, which regulate legitimate competition for social and economic resources that convey status. Societies with stronger institutions and rule of law provide more safety and security for economic and social resources, reducing the costs of ignoring challenges and competition, as well as reducing the necessity of retaliation for males to maintain status (Archer, 2009). More relevant for male adolescents, strong rule of law creates conditions under which resources, such as access to heterosexual relationships, allies (peer network), and popularity, can be achieved without the use of physical aggression (Geary et al., 2003; Pellegrini, 2008). Thus one would expect greater sex differences in societies characterized by, for example, unequal or poorer socioeconomic conditions and weak rule of law. We note that according to sexual selection theory, most cross‐cultural variation in sex differences in physical aggression results from varying levels among *males* rather than among *female*s. In contrast, cross‐cultural differences in female competition for access to males, e.g., due to more or less rigid control over pre‐marital sex, are expected to affect mainly indirect, non‐physical aggression against other females (Campbell, 2013; Vaillancourt, 2013). However, prior research has shown that rates of male and female violence tend to covary (Campbell, 1999), suggesting that to some extent the forces that influence levels of male physical aggression also influence female levels.

To summarize, both social role and sexual selection theories predict that that young males are more likely to use physical aggression against same‐sex competitors than females. However, the two theories lead to different hypotheses about the processes that cause variation in the size of these sex differences between societies. Social role theory argues that societal expectations regarding stereotypical gender roles drive sex differences in violent competition (Wood & Eagly, 2010, 2012). Specifically, the greater the degree of gender inequality within society, the greater sex differences in adolescent violence and aggression will be. Sexual selection theorists expect that the magnitude of sex differences in violence varies with environmental conditions that increase the costs of using direct physical aggression for competition and securing the resources important to reproductive success (Archer, 2009). Sexual selection theory predicts that environmental conditions characterized by greater security and more equal distribution of resources (e.g., lower income inequality, stronger rule of law) will be associated with smaller sex differences in physical aggression.

While there are cross‐cultural evaluations of competing explanations of sex differences in a range of psychological and social behavioral characteristics, limited evidence exists for sex differences in adolescent violence and aggression. The only study that has directly tested these hypotheses relies on a sample from a single country, and measuring gender inequality variation using parental background may not adequately reflect the child's exposure to stereotypical gender socialization (Nivette et al., 2014). Among studies that have investigated variability in sex differences regarding other characteristics or behavior, the sample of societies is often limited primarily to western, highly developed societies where variation in gender polarization and the scarcity of resources is minimal (see Archer, 2004; McCrae & Terracciano, 2005; Schmitt & Rohde, 2013; cf. Campbell, 1995).

### 1.1 Hypotheses

In filling the aforementioned research gaps, this study aims to make two contributions to the understanding of sex differences in physical aggression among adolescents: 1) we describe, for the first time, the prevalence of adolescent physical aggression, as measured by frequent fighting among peers, in sixty‐three low‐ and middle‐income countries (LMICs), focusing explicitly on sex differences and 2) we assess variation in sex differences in physical aggression between these countries and test to what extent sex differences vary in accordance with social role and sexual selection theories.

Specifically, we examine four hypotheses:

1. The *prevalence* of frequent fighting varies significantly across low‐middle income countries.
2. *Sex differences* in frequent fighting vary significantly across low‐ and middle‐income countries.
3. Societal gender inequality explains variation in the sex difference in frequent fighting (social role theory).
4. The provision of security with regards to resources explains variation in the sex difference in frequent fighting (prediction derived from sexual selection theory).

## 2 METHODS

### 2.1 Sample

This study used data from the World Health Organization (WHO) Global School‐based Student Health Survey (GSHS).^1^ The GSHS is a coordinated cross‐national study that assesses a range of risk and protective factors for social and health outcomes among young people aged 13–17 years. According to the GSHS core questionnaire, participation in the survey was voluntary and respondents were ensured their answers would be kept private and there would be no consequences for refusal. We included GSHS data from low‐ and middle‐income countries as defined according to the World Bank (2017) classification, including both lower‐middle‐ and upper‐middle‐income countries. Given that countries can move from middle‐ to upper‐income, we matched the income classification according to the year of the survey(s). We excluded data from two very small countries (Montserrat and Niue) because results were very unstable due to small sample sizes. This resulted in data from 63 countries that conducted surveys on participation in fights between 2003 and 2013. For the purpose of our analyses, we pool data from different years in the same country, resulting in larger sample sizes for some countries.^2^

We restrict our analyses to 12–15 years old because there were very few children surveyed under 12 years old, and in some countries the precise age above 15 years was unclear (e.g., the next age category was 16+). Across the 63 countries, there were a total of 247,909 participants in this age range who had information recorded for the outcome and sex, and who form the maximum sample. On average, there were 3,935 respondents per country (range = 701–22,954). With weighting, the data represent approximately 51 million individuals aged 12–15 years old. Due to missing values for macro‐level indicators, namely gender inequality, the sample used for analyses of variations in sex differences across societies (research questions 3 and 4) is reduced to 52 countries consisting of 222,547 youth.

### 2.2 Measures

#### 2.2.1 Frequent fighting

GSHS participants were asked the following question about participation in fights: “During the past 12 months, how many times were you in a physical fight?”, with ordered categorical response options ranging from “0 times,” “1 time,” “2–3 times,” up to “12 or more times.” The question was preceded by text stating “The next question asks about physical fights. A physical fight occurs when two or more students of about the same strength or power choose to fight each other.” Therefore, GSHS focuses on fights that occur between similar students, regardless of who initiates the fight and the sex of participants, and therefore excludes violence such as robbery, bullying, and sexual assault.

We define *frequent fighting* as self‐reported involvement in four or more fights in the previous 12 months. This approach is similar to previous research that has measured and evaluated frequent fighting in cross‐national surveys (Elgar et al., 2015; Pickett et al., 2013). The cut‐off point reflects more habitual adolescent aggression while excluding occasional or random physical infractions. Single incidents or occasional fighting can be common among youth, whereas frequent fighting has been associated with other types of violence, weapons carrying, substance use, and conduct disorders (see e.g., Smith‐Khuri, Iachan, Scheidt, Overpeck, Gabhainn et al., 2004; Swahn, Bossarte, Palmier, Yao, & van Dulmen, 2013). As shown below, our measure resulted in about 9% of the population being considered “frequent fighters.”^3^

#### 2.2.2 Measures of country‐level explanatory variables

Two country‐level variables were selected that reflect key theoretical concepts relevant to variation in sex differences. To represent the degree of gender polarization in a society as emphasized by social role theory, we used the Gender Inequality Index (GII) produced by the United Nations Development Programme (UNDP, n.d.). The measure combines indicators of the maternal mortality ratio, adolescent fertility rate, female political representation, female secondary and tertiary educational attainment, and female labor market participation rate (see United Nations Development Programme [UNDP], 2013, Technical Note 3). The index ranges from 0 to 1, where higher scores indicate greater gender disparities across the measured dimensions.^4^ We used this measure because it captures disadvantages women face in health, education, and the labor market, as well as values and attitudes to women's roles and thus reflects a valid proxy for societal gender inequality (see Else‐Quest, Hyde, & Linn, 2010:115; Nivette et al., 2014). We averaged GII over 2005–2014 for each country and z‐standardized the measure to have a mean of zero and standard deviation of 1.

One of the key theoretical concepts relevant to variation in sex differences according to sexual selection theory is security of and access to resources that facilitate reproductive success. Previously, this concept has been operationalized using rule of law, income inequality, GDP per capita, and the sex ratio (see Archer, 2009; Schmitt & Rohde, 2013). Here we use a measure of a society's capacity to provide security and protect an individual's access to resources that are associated with power and reproductive success: the World Bank's Rule of Law Index, which is a composite indicator that captures confidence in law and legal agents, as well as the quality of enforcement, justice institutions, and protection of rights (World Bank, 2017). We averaged the Rule of Law Index over 2005–2015 for each country and z‐standardized the measure. The GII and Rule of Law Index are only moderately correlated at the country level (*n* = 52, *r* = −0.29). Thus we can be relatively confident that the two variables reflect different underlying constructs of gender polarization and quality of governance, respectively. As additional analyses, we also investigate whether sex differences in frequent fighting vary according to country‐level income inequality (see Robustness checks section).

### 2.3 Statistical analyses

Our analyses proceeded in four stages. First, we examined the prevalence of frequent fighting in each country in survey‐weighted models. Second, we examined sex differences in frequent fighting. Odds ratios for the sex difference in each country were calculated with frequent fighting as the outcome and sex as the predictor variable. Third, we tested the statistical significance of the variability in the prevalence of frequent fighting and the sex‐fighting relationship in random‐intercept and random‐slope multilevel models.^5^ Fourth, country‐level variables were entered into the multilevel models to examine possible explanations for variation in the sex‐fighting association.^6^ All multilevel models include year fixed effects to account for time trends (Wooldridge, 2002). Analyses were conducted using Stata 14 (StataCorp, 2015).

## 3 RESULTS

**Figure 1:**
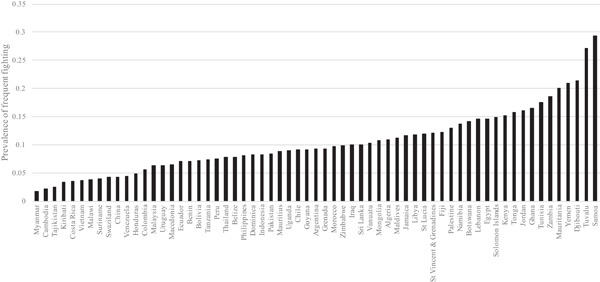
Prevalence of frequent fighting for 63 LMICs (ascending order)

Table 1 shows the frequency of fighting “in the previous 12 months” across all participants aged 12–15 years old in 63 countries. The vast majority of children (roughly 62%) reported no involvement in fights, with a further 29% reporting fighting between 1 and 3 times in the previous 12 months. Those fighting four or more times in the previous 12 months constitute about nine percent of the overall sample. Figure 1 shows the unweighted prevalence of frequent fighting for the 63 countries in the dataset. The country with the highest self‐reported prevalence of adolescent frequent fighting is Samoa (29%), followed by Tuvalu (27%), Djibouti (21%), Yemen (21%), and Mauritania (20%). The countries with the lowest prevalence of frequent fighting were Myanmar and Cambodia (both 2%) and Tajikistan (3%).

**Table 1:** Survey weighted frequency of fighting in previous twelve months by sex

	Female (%)^a^	Male (%)^b^	Total (%)^c^
0 times	72.01	51.11	61.65
1 time	14.27	20.80	17.48
2 or 3 times	8.24	15.15	11.67
4 or 5 times	2.20	5.28	3.74
6 or 7 times	0.99	2.18	1.58
8 or 9 times	0.38	1.06	0.73
10 or 11 times	0.24	0.85	0.54
12+ times	1.67	3.57	2.61
Total	100	100	100

^a^ Base *n* = 130,903; design df = 1,827; population size = 25,641,301.

^b^ Base *n* = 117,006; design df = 1,818; population size = 24,991,349.

^c^ Base *n* = 250,469; design df = 1,950; population size = 51,084,280.

**Figure 2:**
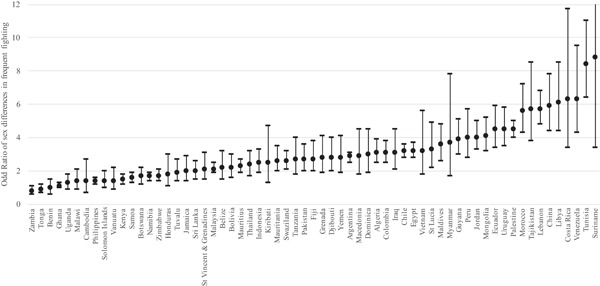
Odds ratios and 95% confidence intervals of sex differences in frequent fighting in 63 low‐ and middle‐income countries (ascending order). Note: y‐axis (odds ratios) truncated at 12 for presentation

Figure 2 shows the extent to which males are more likely to be frequent fighters than females in each of the 63 GSHS countries. Sex differences in frequent fighting are expressed as an odds ratio. In the Online Supplement, Table S1 shows the numeric values for the odds ratio for each country and region as defined by the WHO.^7^ Figure 2 indicates substantial variation between countries. In some countries the sex difference is large—for example in Costa Rica the odds of males being involved in frequent fights are more than six times the odds for females. By contrast, we find that in six countries the odds ratios are around 1.0, meaning that males and females are similarly likely to be involved in frequent fighting (e.g. Zambia, Tonga, Benin, Ghana).

Figures 1 and 2 show substantial variation in both the prevalence of frequent fighting (Figure 1) and the extent of sex differences in frequent fighting (Figure 2). These figures inform the answers to both questions 1 and 2. To further examine variation in frequent fighting, we ran an “empty” multilevel model with data from all 63 countries (247,909 respondents) in order to assess the extent of variation in frequent fighting between countries. This is a model with no explanatory variables in other than t‐1 time‐dummies. Doing so shows 14.42% of variation in frequent fighting is between countries (see Table 2, Model 1), meaning that 85.58% of variation in frequent fighting is within countries. Additionally, we explored whether variation in frequent fighting across countries was different for females and males by running “empty” models for each sex separately. Interestingly, we find that variation in female frequent fighting across countries is greater than variation in male frequent fighting: 20.23% of variation in female fighting is between countries whereas 11.92% of male fighting is between countries (results available in Online Supplement, Table S2).

**Table 2:** Multilevel models for frequent fighting and sex in 63 countries

	Model 1 Empty model			Model 2 Random intercept			Model 3 Random slope		
	OR	95% CI	*p*	OR	95% CI	*p*	OR	95% CI	*p*
Intercept	0.23	(0.18, 0.29)	0.000	0.14	(0.11, 0.17)	0.000	0.13	(0.10, 0.17)	0.000
Sex				2.68	(2.60, 2.76)	0.000	2.68	(2.35, 3.05)	0.000
Random effects									
Intercept variance	0.55	(0.38, 0.80)		0.56	(0.39, 0.80)		0.77	(0.53, 1.12)	
L1 coeff. variance							0.25	(0.18, 0.37)	
Cov (coefficient)							−0.25	(−0.39, −0.11)	
ICC	14.42%	(10.47, 19.53)		14.47%	(10.51, 19.59)		19.01%	(13.98, 25.32)	
Log likelihood		−73771.19			−71461.69			−71036.96	
Likelihood ratio test				M2 vs. M1: χ^2^ (1 df): 4619.01; *p* < 0.001			M3 vs. M2: *χ^2^ (*2 df): 849.46; *p* < 0.001		

All models include t‐1 dummies for year to account for time‐trends (not tabled).

*n*‐sizes for all models. Pupil level (*n* = 247,909), average pupils per country (*n* = 3,935), countries (*n* = 63).

In Model 2 of Table 2 we introduce the variable indicating the sex of respondents. Model 2 shows that on average across all countries and adjusted for time‐trends, adolescent males in these 63 LMICs had more than twice the odds (OR = 2.68; 95% CI = 2.60–2.76) of being involved in frequent fights than females. This is in line with a large body of literature on overall male‐female differences in physical aggression and violence (e.g., Archer, 2009).

Model 2 in Table 2 is based on a random intercept model which assumes the relationship of sex to fighting is “fixed” between countries (i.e., a fixed slope). In contrast, Model 3 is a random slope model, which allows the association of sex with frequent fighting to vary between countries. A likelihood ratio test comparing Models 2 and 3 shows that in Model 3, allowing the relationship between sex and frequent fighting to vary by country fits the data better than the random intercept model (likelihood ratio test *χ* ^2^ = 849.46, *df* = 2, *p *< 0.001). From this, we conclude that there is statistically significant variation in the association of sex with frequent fighting between countries.

In Model 3 the covariance between the intercept (prevalence of frequent fighting for females) and slope (sex difference), reveals an interesting pattern. In this model, the covariance between intercept and slope is negative (covariance = −0.25, 95%CIs = −0.39 to −0.11) meaning that in countries with a higher female prevalence of frequent fighting, the association between sex and frequent fighting decreases, conditional on time trends. In other words, countries with higher female prevalence rates have smaller sex differences (odds ratios) in frequent fighting (for a visualization, we produced a scatterplot depicting the relationship between the predicted random intercepts and slopes, see Figure S1 in the Online Supplement).

Next we introduced country‐level variables indicating social role and sexual selection predictions, respectively, to explain between‐country variation. Rather than over‐fit the model, we fitted one variable at a time and report the results below (Tables 3 and 4). This strategy allowed us to assess whether or not indicators of gender polarization as measured by the Gender Inequality Index (GII) or predictions based on sexual selection theory, as measured by the Rule of Law Index, moderate the sex‐fighting relationship noted above. We used cross‐level interactions between country‐level predictors and sex to explain why sex differences in frequent fighting (i.e., slope variance) might vary across countries (Snijders & Bosker, 2012). Due to missing country‐level data, the sample size for these models is reduced to 52 countries consisting of 222,547 youth.

**Table 3:** Multilevel logistic regression models for prevalence of frequent fighting, average gender inequality index and sex

	Model 1 GII Empty model			Model 2 GII Random intercept			Model 3 GII Random intercept			Model 4 GII Random slope			Model 5 GII Cross‐level interaction		
	OR	95% CI	*p*	OR	95% CI	*p*	OR	95% CI	*p*	OR	95% CI	*p*	OR	95% CI	*p*
Intercept	0.20	(0.16, 0.26)	0.000	0.19	(0.15, 0.25)	0.000	0.11	(0.09, 0.15)	0.000	0.11	(0.08, 0.14)	0.000	0.10	(0.08, 0.13)	0.000
Sex							2.63	(2.55, 2.72)	0.000	2.74	(2.36, 3.18)	0.000	3.01	(2.62, 3.46)	0.000
Country level: Av. GII				1.17	(0.98, 1.40)	0.079	1.16	(0.97, 1.39)	0.095	1.12	(0.91, 1.37)	0.270	1.37	(1.12, 1.68)	0.002
Cross‐level interaction													0.78	(0.69, 0.88)	0.000
Random effects															
Intercept variance	0.52	(0.34, 0.78)		0.49	(0.32, 0.74)		0.49	(0.32, 0.73)		0.66	(0.43, 1.02)		0.61	(0.40, 0.93)	
L1 coeff. variance										0.27	(0.18, 0.42)		0.20	(0.13, 0.31)	
Cov (coefficient)										−0.22	(−0.38, −0.07)		‐0.16	(‐0.28, −0.05)	
ICC	13.62%	(9.48, 19.18)		12.92%	(8.96, 18.28)		12.89%	(8.93, 18.24)		16.69%	(11.45, 23.70)		15.69%	(10.95, 21.96)	
Log likelihood		−63899.90			−63898.40			−61998.70			61628.99			−61621.94	
Likelihood ratio test				M2 vs. M1: *χ* ^2^ (1 df):3.00; *p* < 0.10			M3 vs. M2: *χ* ^2^ (1 df): 3799.40; *p* < 0.001			M4 vs. M3: *χ* ^2^ (2 df): 739.43; *p* < 0.001			M5 vs. M4: χ^2^ (1 df): 14.10; *p* < 0.001		

GII is the average Gender Inequality Index for 2005–2015, z‐standardized with a mean of zero and standard deviation of 1.

*n*‐sizes for all models. Pupil level (*n* = 222,547), average pupils per country (*n* = 4,280), countries (*n* = 52).

All models include t‐1 dummies for year to account for time‐trends (not tabled).

**Table 4:** Multilevel logistic regression models for frequent fighting, average rule of law index and sex

	Model 1 Rule of Law Random intercept			Model 2 Rule of Law Random intercept			Model 3 Rule of Law Random slope			Model 4 Rule of Law Cross‐level interaction		
	OR	95% CI	*p*	OR	95% CI	*p*	OR	95% CI	*p*	OR	95% CI	*p*
Intercept	0.22	(0.17, 0.29)	0.000	0.13	(0.10, 0.17)	0.000	0.12	(0.09, 0.16)	0.000	0.12	(0.09, 0.17)	0.000
Sex				2.63	(2.55, 2.72)	0.000	2.74	(2.36, 3.19)	0.000	2.70	(2.31, 3.16)	0.000
Country level: Av. Rule of Law	1.28	(1.03, 1.59)	0.024	1.28	(1.04, 1.59)	0.022	1.26	(1.02, 1.56)	0.031	1.32	(1.02, 1.70)	0.037
Cross‐level interaction										0.95	(0.81, 1.13)	0.570
Random effects												
Intercept variance	0.47	(0.31, 0.71)		0.47	(0.31, 0.70)		0.67	(0.45, 1.01)		0.67	(0.45, 1.01)	
L1 coeff. variance							0.27	(0.18, 0.42)		0.27	(0.18, 0.41)	
Cov (coefficient)							−0.25	(‐0.40, −0.10)		−0.25	(−0.39, −0.10)	
ICC	12.57%	(8.72, 17.78)		12.43%	(8.62, 17.61)		16.98%	(11.95, 23.56)		16.96%	(11.94, 23.52)	
Log likelihood		−63897.45			−61997.52			−61627.35			−61627.19	
Likelihood ratio test	M1 vs. Empty model *χ* ^2^ (1 df): 4.89; *p* < 0.05			M3 vs. M2: *χ* ^2^ (1 df): 3799.86; *p* < 0.001			M4 vs. M3: *χ* ^2^ (2 df): 740.34; *p* < 0.001			M5 vs. M4: *χ* ^2^ (1 df): 0.32; *p* = 0.571		

Rule of Law is the average Rule of Law Index score for 2005–2015, z‐standardised with a mean of zero and standard deviation of 1.

n‐sizes for all models. Pupil level (*n* = 222,547), average pupils per country (*n* = 4,280), countries (*n* = 52).

All models include t‐1 dummies for year to account for time‐trends (not tabled).

**Figure 3:**
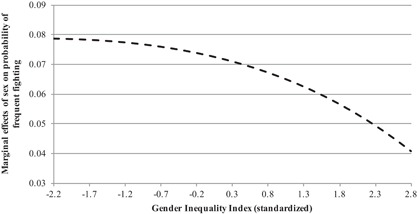
Estimated marginal effect of sex on frequent fighting by country‐level gender inequality

Table 3 reports the results for gender inequality using the GII. Overall, we see that there is no direct association between GII and fighting (e.g., Model 2, OR = 1.17, 95%CI = 0.98–1.40). Model 5 in Table 3 reports the estimates for the cross‐level interaction between GII and sex. The main effect for GII suggests a positive relationship between GII and female fighting: the odds ratio for the GII measure when sex is coded zero (female), is 1.37 (95%CI = 1.12–1.68, *p *= 0.002). This means that as GII increases, females are more likely to engage in frequent fighting. The cross‐level interaction in that model is also significant (OR = 0.78, 95%CI = 0.69–0.88, *p *= 0.000), and suggests that as GII increases, the ratio between males and females in frequent fighting decreases. To better visualize this relationship, we estimated the average marginal relationship between sex and frequent fighting by level of GII (see Figure 3). Results are estimated using the fixed portion only and holding all other variables at their means. Figure 3 shows that the relationship between sex and fighting decreases as societal gender inequality gradually increases.

Next we assessed the predictions made by sexual selection theory (research question 4). Estimates for the Rule of Law Index are reported in Table 4. Average Rule of Law was significantly positively related to adolescent frequent fighting (Model 1 OR = 1.28, 95%CI = 1.03–1.59, *p *= 0.024), meaning adolescents in countries with stronger rule of law tend to report engaging in more frequent fighting. This finding is consistent across models but is counter‐intuitive. In Model 4, the inclusion of the cross‐level interaction is not significant and does not significantly improve model fit (Model 4 *χ^2^* (1 df) = 0.32, *p *= 0.571).

### 3.1 Robustness checks

Our measure of frequent fighting was designed to capture regular fighting without casting the net too wide and including all fights in the past year. Nonetheless, we ran a series of robustness checks on the consistency of results at different cut‐off points of physical aggression. We re‐estimated models using cut‐off points at one or more physical fights (i.e., any fighting) and 2‐3+ physical fights (“intermittent to frequent fighting”). We focus on these categories because at higher levels, the number of students involved drops significantly.

In addition, country‐level rule of law reflects one of several environmental dimensions that influence male competition according to sexual selection theorists (security of resources). However, sexual selection theory also predicts that the unequal distribution and scarcity of resources can increase intrasexual competition among males (Archer, 2009). In light of this, we re‐estimated all models replacing country‐level rule of law with a measure of income inequality (the GINI Index)^8^ for each of the different possible cut‐off points for physical fighting (i.e., 1+, 2–3+, 4+). The results show that income inequality has no direct or conditional effect on the likelihood of physical aggression or the size of the sex difference (see Table S9–S11 in the Online Supplement), with one exception. Model 5 in Table S10 in the Online Supplement shows that the sex difference in any fighting (1 or more physical fights) does vary significantly according to country‐level income inequality, however, the effect is in the opposite direction as expected (OR = 0.87, 95%CI = 0.76–0.99, *p *= 0.033). This means that as income inequality increases, the ratio between male and female fighting decreases (see Figure S2 in Online Supplement).

The robustness checks indicate that our results are largely robust to different cut‐offs for prevalence of physical aggression and additional measures of resource distribution (i.e., GINI Index) as derived from sexual selection theory (see the Online Supplement for full results). Namely, whatever the specification, we still observe: (i) substantial variation in both male and female prevalence; (ii) no or contradictory variation in sex differences by country‐level rule of law and income inequality; and (iii) significant variation in sex differences by country‐level gender inequality, wherein the strength of the relationship between sex and physical aggression decreases at higher levels of gender inequality. Overall our results suggest that overall rates of physical fighting are primarily driven by male involvement, as evidenced by the much higher overall prevalence of male violence in nearly all countries where the GSHS survey was conducted. But we also show that there is substantial cross‐national variation in female violence and under some rare conditions, female rates of frequent physical fighting equal that of males.

## 4 DISCUSSION

Previous research shows that adolescent males are more likely to engage in physical fights than females (Archer, 2004). However, few studies have examined cross‐cultural variation in sex differences, particularly among low‐ and middle‐income countries. The current study examined the prevalence and variation in sex differences in frequent fighting between equal partners in 63 low‐ and middle‐income countries. Specifically, we tested competing hypotheses derived from social role and sexual selection theories using indicators of gender inequality, rule of law, and income inequality. The results show that sex differences in physical aggression vary significantly across countries. Further, we found little evidence to support predictions derived from sexual selection or social role theories in relation to cross‐cultural variation, and indeed our results showed that sex differences actually *decrease* as gender inequality increases.

This study advances our knowledge of adolescent aggression in several ways. To our knowledge, this is the largest study looking at cross‐national variation in adolescent physical aggression in LMICs. Our results show that there is significant variation in the prevalence of frequent fighting across countries, ranging from 2% (Myanmar) to 29% (Samoa).

Furthermore, in line with previous research, adolescent males are significantly more likely to engage in frequent fighting than females, with few exceptions (e.g., Archer, 2004; Bettencourt & Miller, 1996). For the full sample of 63 countries, males were 2.68 times more likely to report frequent fighting in the past 12 months than females. But when we looked at female involvement in frequent fighting, we found this varied substantially across societies, perhaps more than evolutionary perspectives would expect (ranging from 0.07% [Tajikistan] to 25% [Samoa]). The cross‐national variation in frequent fighting was also greater among females than males (20.23% and 11.92%, respectively). Furthermore male and female frequent fighting covary (*r *= 0.69), suggesting that some forces that drive physical aggression are not sex‐specific.

This study also finds that contrary to expectations derived from social role theory, sex differences in physical aggression decreased as societal gender inequality increased. Several explanations may account for the finding that sex differences were reduced in countries with high levels of gender inequality. First, there may be a confounding factor in which societies with very high levels of gender inequality typically have institutional arrangements that strictly limit male adolescent competition for females through separate schooling, an emphasis on segregated routine activities, and severe stigma associated with pre‐marital sex (United Nations Development Programme [UNDP], 2016; Wiseman, 2008). This segregation of adolescent male and female life spheres may limit the kinds of male formidability competitions that are likely one of the motivational roots of fighting among adolescent males.

Second, evolutionary perspectives may also provide an explanation for higher levels of female physical aggression in high gender inequality societies. In her overview of female aggression, Campbell (1995, 1999) argued that females are more likely to engage in aggressive behavior when access to “successful” males is scarce. Geary, Winegard, and Winegard (2014) point out that female‐female competition over access and choice of mates is particularly influenced by the structure of marital and kin arrangements in a society. In traditional societies where polygynous marriage arrangements are more common, females, including adolescents, may be under more pressure to compete for status and resources and thus more likely to employ direct and indirect aggressive tactics to succeed. Indeed, we find that the smallest sex differences tend to be found in Sub‐Saharan African countries (average odds ratio = 1.8), where polygynous marriages are more prevalent (Omariba & Boyle, 2007) and GII is on average higher compared to other regions in the current sample. Also, societies vary in the extent to which adolescent girls engage in transactional sex, the exchange of sexual services for money or gifts (e.g., Fredlund, Svensson, Svedin, Priebe, & Wadsby, 2013). Evidence suggests that several sub‐Saharan countries likely have an elevated prevalence of transactional sex among adolescent girls (Chatterji, Murray, London, & Anglewicz, 2005). These arrangements may contribute to higher intra‐sexual competition for sexual partners among adolescent girls, and hence contribute to a higher prevalence of frequent fighting. Notably, under these same circumstances males are also more likely to compete for resources for sexual success (Schmitt & Rohde, 2013).

With regard to sexual selection theory, we did not find evidence that sex differences in frequent fighting varied according to societal rule of law. Interestingly, we find that frequent fighting is greater in societies with stronger rule of law. One possible explanation is that in societies where the rule of law is weak, more serious violence is prevalent in the community. Under these conditions, students may be less likely to enter into physical fights in fear of more serious retaliation by their opponent with a weapon or by a gang. In other words, the consequences of students fighting are potentially more serious when the criminal justice system is absent and community violence is rampant. However, more research is needed to understand this counterintuitive finding.

### 4.1 Limitations and future research

There are several limitations to this study. First, although the data reflect a sample of adolescents from countries that are typically underrepresented in aggression and violence research, the sample of countries is nevertheless non‐random and therefore limits generalizability. However, given the push among international organizations such as the United Nations and World Health Organization to increase research and prevention knowledge outside developed western societies, this study contributes to this growing effort to understand adolescent aggression on a global level.

Second, the GSHS data only reflect a snapshot of adolescents in a given society, and thus results reported here may be attributable in part to age‐period‐cohort effects, particularly in countries where many years of data are pooled together. One counter to the “period” effect within the sample data is that all models include year dummies as covariates. In addition, the macro‐level factors used in this study are broad indicators of institutional and social structures and aggregate adult behavior. Thus these variables may not accurately reflect the lived experiences of adolescents in a school context. Future research should explore further variations in sex differences using alternative measures of gender polarization (e.g., female access to education, Standardized Index of Gender Equality) and the security or scarcity of resources for reproductive success (e.g., GDP per capita, access to education). Nevertheless, to our knowledge, this is one of the first attempts to open up debate on the extent and possible explanations for sex differences in LMICs using large‐scale data.

Third, our focus on four or more physical fights (i.e., frequent fighting) as an indicator of physical aggression ensured that we captured more serious, habitual behavior rather than sporadic, occasional encounters. While this measure has been used in previous cross‐country studies and validated as a reflection of broader violence and misconduct (Elgar et al., 2015; Smith‐Khuri et al., 2004), it is nevertheless unknown to what extent frequent fighting reflects physical aggression in the wider population. Furthermore, the definition of physical fighting in the GSHS as “two or more students of about the same strength or power choose to fight each other” may introduce response bias, as there is some question as to how “power” would be rendered in translation, and whether students involved with those stronger or more “powerful” would be accurately captured. Similarly, the specification of “choosing” to fight might cause response bias as fights might be involuntary.

Finally, driven in part by the number of countries with available data, we limited the predictors in the model to sex and selected country‐level indicators to focus on describing and explaining variations in the sex gap across societies. By limiting the model to these measures, we may be omitting theoretically important predictors of adolescent physical aggression. Our primary aim was not to explain average levels of adolescent aggression, but to examine the varying sex differences in aggression across societies.

## 5 CONCLUSION

Male sex is considered one of the strongest and most robust correlates of crime and delinquency across time and place (Wright, Beaver, & Ellis, 2009). However, most studies in behavioral science have been conducted with “WEIRD” populations—in Western, Educated, Industrialized, Rich, Democracies—which may be “among the least representative populations one could find for generalizing about humans” (Henrich, Heine, and Norenzayan, 2010, p. 61). Our finding that sex‐differences in fighting vary considerably across LMICs suggests that most current evidence on correlates of antisocial behavior, derived mainly from high‐income countries, may not apply outside these contexts. Although the cross‐national replicability of risk factors for antisocial behavior has been demonstrated for example between England and the United States (Farrington & Loeber, 1999; see also Wright et al., 2009), the fact that a person's sex has such different implications for their involvement in fighting in other contexts implies cross‐national replicability of risk factors certainly should not be taken for granted. Hence, the body of risk factor research that has informed developmental theories of antisocial behavior, and prevention strategies based on those theories, may require re‐evaluating in LMICs, where most of the world's population lives.

## Supporting information

Additional Supporting Information may be found online in the supporting information tab for this article.

**Table S1**. GSHS survey characteristics in 63 low‐ and middle‐income countries
**Table S2**. Multilevel models for frequent fighting by sex in 63 countries
**Table S3**. Multilevel models for any fighting (1+ times) and sex in 63 countries
**Table S4**. Multilevel logistic regression models for prevalence of any fighting (1+ times), average gender inequality index and sex
**Table S5**. Multilevel logistic regression models for any fighting (1+ times), average rule of law index and sex
**Table S6**. Multilevel models for intermittent to frequent fighting (2‐3+ times) and sex in 63 countries
**Table S7**. Multilevel logistic regression models for prevalence of intermittent to frequent fighting (2‐3+ times), average gender inequality index and sex
**Table S8**. Multilevel logistic regression models for intermittent to frequent fighting (2‐3+ times), average rule of law index and sex
**Table S9**. Multilevel logistic regression models for frequent fighting, income inequality, and sex
**Table S10**. Multilevel logistic regression models for any fighting (1+ times), income inequality, and sex
**Table S11**. Multilevel logistic regression models for any fighting (2‐3+ times), income inequality, and sex
**Figure S1**. Bivariate distribution of random slope and intercept (n=63 countries)
**Figure S2**. Estimated marginal effect of sex on any fighting (1+ times) by country‐level income inequality (*n *= 51 countries)

## ENDNOTES

1 Data are available online at: http://www.who.int/ncds/surveillance/gshs/datasets/en/.

2 In all but three countries, two‐stage cluster sampling was used: first, schools were selected with a probability proportional to enrolment size; second, school classes of the target age group were randomly selected, and all students in the selected classes were eligible to participate. In Ecuador (Zamora) a census was used to produce data representative of all students in grades 8, 9, 10, and the Freshman year.

3 The original GSHS English questionnaire was translated into local languages and back translated during the development of surveys in each country. In a separate process, we translated the key question on fights back to English from Spanish, French, Chinese, Portuguese, Russian, and Arabic. One difference we observed in conducting these back translations was that (unlike in English, Spanish, Chinese, Portuguese, and Russian and Arabic) in French there was reference to participation in “quarrels” as well as “physical fights,” broadening the scope of the behaviors captured by questionnaires applied in French.

4 Data are available at: https://data.undp.org/dataset/GII-Gender-Inequality-Index-value/bh77-rzbn.

5 The generalized form of the multilevel equation for random slopes (random coefficients) and two levels is: $y_{ij}=b0+{b1}_{j}{xl}_{ij}+{b2}_{z}{x2}_{j}+\text{…}BkXk+{U0}_{j}+e_{ij}$ where *y* is an outcome that varies for individuals *i* and countries *j* and b0 is the overall intercept. b1*_j_*x1*_ij_* is an individual level covariate, the slope of which is allowed to vary between countries (hence b1*_j_*). x2*j* is a country‐level measure, hence only having the subscript *j*. U0*_j_* represents the residual variation (error) between countries and *e_ij_* the residual variation (error) within countries.

6 With a maximum sample of 63 countries, we are limited in the number of country level measures we can include. Rules of thumb used typically suggest some ratio of cases to variables, but the real issue regards statistical power and estimation of standard errors (Maas & Hox, 2005).

7 The odds ratios reported here are based on unweighted data, because logistic regression models accounting for weighting could not be run for all countries. For the countries for which both weighted and unweighted odds ratios could be calculated, the estimates were extremely similar (*r *= 0.94 for the pairs of 54 weighted and unweighted odds ratios).

8 Income inequality was measured using the GINI Index, and data are available online from the World Bank: https://data.worldbank.org/indicator/SI.POV.GINI. Estimates from 2014 or the most recent year were used, resulting in a sample of 51 countries with data available. The GINI Index ranges from 0 (perfect equality) to 1 (perfect inequality). The Rule of Law and GINI Index were preferred over other measures of country‐level resource distribution (e.g., GDP per capita, education) because the former are less strongly correlated with gender inequality and thus more conceptually distinct.
