# Supplementary material for: Sex differences in adolescent physical aggression: Evidence from sixty‐three low‐and middle‐income countries
Source: Aggress Behav. 2018 Oct 3;45(1):82–92. doi: 10.1002/ab.21799 (PMC6950221; doi:10.1002/ab.21799)
Supplement: Supplementary file 1 — Table S1. GSHS survey characteristics in 63 low‐ and middle‐income countries Table S2. Multilevel models for frequent fighting by sex in 63 countries Table S3. Multilevel models for any fighting (1+ times) and sex in 63 countries Table S4. Multilevel logistic regression models for prevalence of any fighting (1+ times), average gender inequality index and sex Table S5. Multilevel logistic regression models for any fighting (1+ times), average rule of law index and sex Table S6. Multilevel models for intermittent to frequent fighting (2‐3+ times) and sex in 63 countries Table S7. Multilevel logistic regression models for prevalence of intermittent to frequent fighting (2‐3+ times), average gender inequality index and sex Table S8. Multilevel logistic regression models for intermittent to frequent fighting (2‐3+ times), average rule of law index and sex Table S9. Multilevel logistic regression models for frequent fighting, income inequality, and sex Table S10. Multilevel logistic regression models for any fighting (1+ times), income inequality, and sex Table S11. Multilevel logistic regression models for any fighting (2‐3+ times), income inequality, and sex Figure S1. Bivariate distribution of random slope and intercept (n=63 countries) Figure S2. Estimated marginal effect of sex on any fighting (1+ times) by country‐level income inequality (n = 51 countries) [file AB-45-82-s001.docx]

**Online Supplement: Sex differences in adolescent physical aggression (Nivette et al.)**

**OS Table 1: GSHS survey characteristics in 63 low- and middle-income countries**

| **Country** | **Survey Year** | **Total** | **Males** | **Females** | **Total** | **Males** | **Females** | **Male-Female** |
| --- | --- | --- | --- | --- | --- | --- | --- | --- |
|  |  | **N** | **N** | **N** | **Prevalence Frequent Fights** | **Prevalence Frequent Fights** | **Prevalence Frequent Fights** | **Odds Ratio** |
| **African Region** |  |  |  |  |  |  |  |  |
| Algeria | 2011 | 3465 | 1583 | 1882 | .11 | .17 | .06 | 3.1 (2.5-3.9) |
| Benin | 2009 | 1168 | 741 | 427 | .07 | .07 | .07 | 1.0 (0.6-1.5) |
| Botswana | 2005 | 1391 | 592 | 799 | .14 | .18 | .11 | 1.7 (1.2-2.2) |
| Ghana | 2007-2012 | 5588 | 2748 | 2840 | .16 | .17 | .15 | 1.1 (1.0-1.3) |
| Kenya | 2003 | 2917 | 1362 | 1555 | .15 | .18 | .13 | 1.5 (1.2-1.8) |
| Malawi | 2009 | 2198 | 1022 | 1176 | .04 | .04 | .03 | 1.4 (0.9-2.1) |
| Mauritania | 2010 | 1281 | 586 | 695 | .20 | .28 | .13 | 2.6 (2.0-3.5) |
| Mauritius | 2007-2011 | 6139 | 2818 | 3321 | .09 | .12 | .06 | 2.3 (1.9-2.7) |
| Namibia | 2004 | 4529 | 2014 | 2515 | .14 | .17 | .11 | 1.7 (1.4-1.9) |
| Swaziland | 2003 | 7734 | 2787 | 4947 | .04 | .07 | .03 | 2.6 (2.1-3.2) |
| Tanzania | 2006 | 1751 | 852 | 899 | .07 | .10 | .04 | 2.7 (1.8-4.0) |
| Uganda | 2003 | 1903 | 914 | 989 | .09 | .10 | .08 | 1.3 (0.9-1.8) |
| Zambia | 2004 | 1335 | 607 | 728 | .18 | .16 | .19 | 0.8 (0.6-1.1) |
| Zimbabwe | 2003 | 3919 | 1599 | 2320 | .10 | .13 | .08 | 1.7 (1.4-2.1) |
| **African Average** |  |  |  |  | **.11** | **.14** | **.09** | **1.8** |
| **Americas** |  |  |  |  |  |  |  |  |
| Argentina | 2007-2012 | 22954 | 10766 | 12188 | .09 | .13 | .05 | 2.9 (2.5-3.1) |
| Belize | 2011 | 1602 | 751 | 851 | .08 | .11 | .05 | 2.2 (1.5-3.2) |
| Bolivia | 2012 | 2823 | 1398 | 1425 | .07 | .10 | .05 | 2.2 (1.6-3.0) |
| Chile | 2004-5 | 14416 | 7073 | 7343 | .09 | .14 | .05 | 3.2 (2.8-3.6) |
| Colombia | 2007 | 8001 | 3531 | 4470 | .06 | .09 | .03 | 3.1 (2.5-3.8) |
| Costa Rica | 2009 | 2266 | 1077 | 1189 | .03 | .06 | .01 | 6.3 (3.4-11.7) |
| Dominica | 2009 | 1289 | 560 | 729 | .08 | .13 | .05 | 3.0 (1.9-4.5) |
| Ecuador | 2007 | 4533 | 2176 | 2357 | .07 | .12 | .03 | 4.5 (3.4-5.9) |
| Grenada | 2008 | 1300 | 564 | 736 | .09 | .14 | .06 | 2.8 (1.9-4.1) |
| Guyana | 2004-10 | 3012 | 1291 | 1721 | .09 | .15 | .04 | 3.9 (3.0-5.1) |
| Honduras | 2012 | 1455 | 690 | 765 | .05 | .06 | .03 | 1.8 (1.1-3.0) |
| Jamaica | 2010 | 1202 | 585 | 617 | .11 | .15 | .08 | 2.0 (1.4-2.9) |
| Peru | 2010 | 2358 | 1144 | 1214 | .08 | .12 | .03 | 4.0 (2.8-5.7) |
| Saint Lucia | 2007 | 1069 | 450 | 619 | .12 | .19 | .07 | 3.3 (2.2-4.9) |
| Saint Vincent and the Grenadines | 2007 | 1201 | 559 | 642 | .12 | .16 | .08 | 2.1 (1.5-3.1) |
| Suriname | 2009 | 1050 | 491 | 559 | .04 | .07 | .01 | 8.8 (3.4-22.5) |
| Uruguay | 2006 | 5762 | 2676 | 3086 | .06 | .11 | .03 | 4.5 (3.5-5.8) |
| Venezuela | 2003 | 3928 | 1747 | 2181 | .04 | .08 | .01 | 6.3 (4.3-9.5) |
| **Americas Average** |  |  |  |  | **.08** | **.12** | **.04** | **3.7** |
| **South-East Asia Region** |  |  |  |  |  |  |  |  |
| Indonesia | 2007 | 2992 | 1414 | 1578 | .08 | .12 | .05 | 2.5 (1.9-3.3) |
| Maldives | 2009 | 1996 | 879 | 1117 | .11 | .18 | .06 | 3.6 (2.6-4.8) |
| Myanmar | 2007 | 2233 | 1074 | 1159 | .02 | .03 | .01 | 3.7 (1.7-7.8) |
| Sri Lanka | 2008 | 2481 | 1076 | 1405 | .10 | .13 | .07 | 2.0 (1.5-2.6) |
| Thailand | 2008 | 2666 | 1307 | 1359 | .08 | .11 | .05 | 2.4 (1.7-3.2) |
| **South-East Asia Average** |  |  |  |  | **.08** | **.11** | **.05** | **2.8** |
| **European Region** |  |  |  |  |  |  |  |  |
| Macedonia | 2007 | 1535 | 761 | 774 | .06 | .09 | .03 | 2.9 (1.8-4.5) |
| Tajikistan | 2006 | 7412 | 3659 | 3753 | .02 | .04 | .01 | 5.7 (3.8-8.5) |
| **European Average** |  |  |  |  | **.04** | **.07** | **.02** | **4.3** |
| **Eastern Mediterranean Region** | |  |  |  |  |  |  |  |
| Djibouti | 2007 | 1020 | 591 | 429 | .21 | .28 | .12 | 2.8 (2.0-4.0) |
| Egypt | 2006-2011 | 7332 | 3663 | 3669 | .15 | .21 | .08 | 3.2 (2.8-3.7) |
| Iraq | 2012 | 1528 | 835 | 693 | .10 | .14 | .05 | 3.1 (2.07-4.5) |
| Jordan | 2004-2007 | 3475 | 1740 | 1735 | .16 | .25 | .07 | 3.1 (2.1-4.5) |
| Lebanon | 2007 | 6484 | 2959 | 3525 | .15 | .25 | .06 | 5.7 (4.8-6.8) |
| Libya | 2007 | 1888 | 788 | 1100 | .12 | .22 | .04 | 6.1 (4.4-8.5) |
| Morocco | 2006 | 4423 | 2183 | 2240 | .10 | .16 | .03 | 5.6 (4.3-7.2) |
| Pakistan | 4981 | 4981 | 3726 | 1255 | .08 | .12 | .04 | 2.7 (2.0-3.6) |
| Tunisia | 2008 | 2534 | 1213 | 1321 | .18 | .31 | .05 | 8.4 (6.4-11.0) |
| Yemen | 2008 | 897 | 531 | 366 | .21 | .27 | .12 | 2.8 (1.9-4.1) |
| Palestine | 2010 | 13606 | 6240 | 7366 | .13 | .21 | .06 | 4.5 (4.0-5.0) |
| **Eastern Mediterranean Average** |  |  |  |  | **.14** | **.22** | **.07** | **4.4** |
| **Western Pacific Region** |  |  |  |  |  |  |  |  |
| Cambodia | 2013 | 1807 | 797 | 1010 | .02 | .03 | .02 | 1.4 (0.7-2.7) |
| China | 2003 | 8463 | 4105 | 4358 | .04 | .07 | .01 | 5.9 (4.4-7.8) |
| Fiji | 2010 | 1493 | 632 | 861 | .12 | .18 | .08 | 2.7 (2.0-3.8) |
| Kiribati | 2011 | 1352 | 566 | 786 | .03 | .05 | .02 | 2.5 (1.3-4.7) |
| Malaysia | 2012 | 16249 | 8326 | 7923 | .06 | .08 | .04 | 2.1 (1.9-2.5) |
| Mongolia | 2013 | 3690 | 1763 | 1927 | .11 | .17 | .05 | 4.1 (3,2-5.2) |
| Philippines | 2003-7 | 11673 | 4785 | 6888 | .08 | .10 | .07 | 1.4 (1.2-1.6) |
| Samoa | 2011 | 2048 | 827 | 1221 | .29 | .34 | .25 | 1.6 (1.3-1.9) |
| Solomon Islands | 2011 | 925 | 460 | 465 | .16 | .12 | .14 | 1.4 (1.0-2.0) |
| Tonga | 2014 | 1906 | 853 | 1053 | .16 | .15 | .16 | 0.9 (0.7-1.2) |
| Tuvalu | 2013 | 701 | 341 | 360 | .27 | .33 | .21 | 1.9 (1.4-2.7) |
| Vanuatu | 2011 | 851 | 356 | 495 | .10 | .12 | .09 | 1.4 (0.9-2.2) |
| Vietnam | 2013 | 1729 | 802 | 927 | .04 | .06 | .02 | 3.2 (1.8-5.6) |
| **Western Pacific Average** |  |  |  |  | **.11** | **.14** | **.09** | **2.3** |
| **All countries** | 2003-13 | 250469 | 117006 | 130903 | **.09** | **.13** | **.06** | **3.2** |

**OS Table 2. Multilevel models for frequent fighting by sex in 63 countries**

|  | **Females** | | | **Males** | | |
| --- | --- | --- | --- | --- | --- | --- |
|  | **OR** | **95% CI** | **p** | **OR** | **95% CI** | **p** |
| Intercept | 0.15 | (0.11, 0.21) | 0.000 | 0.27 | (0.21,0.34) | 0.000 |
| *Random effects* |  |  |  |  |  |  |
| Intercept variance | 0.83 | (0.57,1.21) |  | 0.45 | (0.31,0.65) |  |
| ICC | 20.23% | (14.87, 26.91) |  | 11.92% | (8.51, 16.45) |  |
| Log likelihood |  | -26780.07 |  |  | -44266.52 |  |

Note 1: All models include t-1 dummies for year to account for time-trends (not tabled).

Note 2: n-sizes for female model: pupil level (n=130,903), average pupils per country (n=2,078), countries (n=63).

Note 2: n-sizes for male model: pupil level (n=117,006), average pupils per country (n=1,857), countries (n=63).

**OS Table 3. Multilevel models for any fighting (1+ times) and sex in 63 countries**

|  | **Model 1**  **Empty model** | | | **Model 2**  **Random intercept** | | | **Model 3**  **Random slope** | | |
| --- | --- | --- | --- | --- | --- | --- | --- | --- | --- |
|  | **OR** | **95% CI** | **p** | **OR** | **95% CI** | **p** | **OR** | **95% CI** | **p** |
| *Intercept* | 1.04 | (0.88, 1.24) | 0.638 | 0.67 | (0.56,0.80) | 0.000 | 0.64 | (0.52,0.78) | 0.000 |
| Sex |  |  |  | 2.66 | (2.62,2.71) | 0.000 | 2.57 | (2.28,2.89) | 0.000 |
| *Random effects* | |  |  |  |  |  |  |  |  |
| Intercept variance | 0.33 | (0.23,0.48) |  | 0.36 | (0.25,0.51) |  | 0.49 | (0.34,0.71) |  |
| L1 coeff. variance | |  |  |  |  |  | 0.22 | (0.15,0.32) |  |
| Cov(coefficient) | |  |  |  |  |  | -0.19 | (-0.29,-0.09) |  |
| ICC | 9.23% | (6.65, 12.67) |  | 9.81% | (7.08, 13.43) |  | 13.01% | (9.43, 17.68) |  |
| Log likelihood |  | -157643.63 |  |  | -151333.95 |  |  | -150245.87 |  |
| Likelihood ratio test |  |  |  | M2 vs M1: | | | M3 vs M2: | | |
|  |  |  |  | *χ^2^* (1 df) 12619.36; p<.001 | | | *χ^2^ (*2 df) 2176.15; p<.001 | | |

Note 1: All models include t-1 dummies for year to account for time-trends (not tabled).

Note 2: n-sizes for all models. Pupil level (n=247,909), average pupils per country (n=3,935), countries (n=63).

**OS Table 4: Multilevel logistic regression models for prevalence of any fighting (1+ times), average gender inequality index and sex**

|  | **Model 1 GII**  **Empty model** | | | **Model 2 GII**  **Random intercept** | | | **Model 3 GII**  **Random intercept** | | | **Model 4 GII**  **Random slope** | | | **Model 5 GII**  **Cross-level interaction** | | |
| --- | --- | --- | --- | --- | --- | --- | --- | --- | --- | --- | --- | --- | --- | --- | --- |
|  | **OR** | **95% CI** | **p** | **OR** | **95% CI** | **p** | **OR** | **95% CI** | **p** | **OR** | **95% CI** | **p** | **OR** | **95% CI** | **p** |
| *Intercept* | 0.91 | (0.77, 1.08) | 0.299 | 0.86 | (0.72, 1.02) | 0.074 | 0.55 | (0.46, 0.65) | 0.000 | 0.52 | (0.43, 0.63) | 0.000 | 0.50 | (0.41, 0.60) | 0.000 |
| Sex |  |  |  |  |  |  | 2.64 | (2.59,2.69) | 0.000 | 2.67 | (2.32,3.04) | 0.000 | 2.90 | (2.55,3.29) | 0.000 |
| *Country level: Av. GII* |  |  |  | 1.19 | (1.05,1.35) | 0.007 | 1.18 | (1.04,1.35) | 0.010 | 1.17 | (1.01,1.36) | 0.033 | 1.33 | (1.15,1.53) | 0.000 |
| *Cross-level interaction* | |  |  |  |  |  |  |  |  |  |  |  | 0.80 | (0.71,0.89) | 0.000 |
| *Random effects* |  |  |  |  |  |  |  |  |  |  |  |  |  |  |  |
| Intercept variance | 0.28 | (0.19,0.41) |  | 0.24 | (0.16,0.36) |  | 0.26 | (0.17,0.38) |  | 0.33 | (0.22,0.51) |  | 0.31 | (0.21,0.47) |  |
| L1 coeff. variance |  |  |  |  |  |  |  |  |  | 0.24 | (0.16,0.36) |  | 0.18 | (0.12,0.28) |  |
| Cov(coefficient) |  |  |  |  |  |  |  |  |  | -0.14 | (-0.24,-0.04) |  | -0.10 | (-0.18,-0.03) |  |
| ICC | 7.78% | (5.37, 11.15) |  | 6.87% | (4.72, 9.90) |  | 7.26% | (4.99, 10.43) |  | 9.14% | (6.17, 13.34) |  | 8.67% | (5.96, 12.44) |  |
| Log likelihood |  | -140757.91 |  |  | -140754.53 |  |  | -135218.43 |  |  | -134189.92 |  |  | -134183.16 |  |
| Likelihood ratio test |  |  |  | M2 vs M1: | | | M3 vs M2: | | | M4 vs M3: | | | M5 vs M4: | | |
|  |  |  |  | *χ^2^* (1 df): 6.77; p<.01 | | | *χ^2^* (1 df): 11072.19; p<.001 | | | *χ^2^* (2 df): 2057.02; p<.001 | | | *χ^2^* (1 df): 13.54; p<.001 | | |

Note 1: GII is the average Gender Inequality Index for 2005-2015, z-standardized with a mean of zero and standard deviation of 1.

Note 2: n-sizes for all models. Pupil level (n=222,547), average pupils per country (n=4,280), countries (n=52).

Note 3: All models include t-1 dummies for year to account for time-trends (not tabled).

**OS Table 5: Multilevel logistic regression models for any fighting (1+ times), average rule of law index and sex**

|  | **Model 1 Rule of Law**  **Random intercept** | | | **Model 2 Rule of Law**  **Random intercept** | | | **Model 3 Rule of Law**  **Random slope** | | | **Model 4 Rule of Law**  **Cross-level interaction** | | |
| --- | --- | --- | --- | --- | --- | --- | --- | --- | --- | --- | --- | --- |
|  | **OR** | **95% CI** | **p** | **OR** | **95% CI** | **p** | **OR** | **95% CI** | **p** | **OR** | **95% CI** | **p** |
| *Intercept* | 0.97 | (0.81, 1.17) | 0.772 | 0.62 | (0.52, 0.75) | 0.000 | 0.59 | (0.48, 0.72) | 0.000 | 0.59 | (0.48, 0.73) | 0.000 |
| Sex |  |  |  | 2.64 | (2.59,2.69) | 0.000 | 2.66 | (2.32,3.05) | 0.000 | 2.63 | (2.28,3.05) | 0.000 |
| *Country level: Av. Rule of Law* | 1.18 | (1.01,1.38) | 0.043 | 1.19 | (1.01,1.40) | 0.036 | 1.17 | (1.001,1.37) | 0.049 | 1.19 | (0.98,1.45) | 0.072 |
| *Cross-level interaction* |  |  |  |  |  |  |  |  |  | 0.98 | (0.84,1.14) | 0.752 |
| *Random effects* |  |  |  |  |  |  |  |  |  |  |  |  |
| Intercept variance | 0.26 | (0.17,0.38) |  | 0.27 | (0.18,0.40) |  | 0.39 | (0.26,0.58) |  | 0.39 | (0.26,0.58) |  |
| L1 coeff. variance |  |  |  |  |  |  | 0.24 | (0.16,0.36) |  | 0.24 | (0.16,0.36) |  |
| Cov(coefficient) |  |  |  |  |  |  | -0.18 | (-0.28,-0.08) |  | -0.18 | (-0.28,-0.08) |  |
| ICC | 7.27% | (5.01, 10.43) |  | 7.55% | (5.21, 10.82) |  | 10.52% | (7.30, 14.93) |  | 10.52% | (7.30, 14.92) |  |
| Log likelihood |  | -140755.93 |  |  | -135219.41 |  |  | -134190.21 |  |  | -134190.16 |  |
| Likelihood ratio test | M1 vs. Empty model | | | M3 vs M2: | | | M4 vs M3: | | | M5 vs M4: | | |
|  | *χ^2^* (1 df): 3.97; p<.05 | | | *χ^2^* (1 df): 11073.03; p<.001 | | | *χ^2^* (2 df): 2058.40; p<.001 | | | *χ^2^* (1 df): 0.10; p=.752 | | |

Note 1: Rule of Law is the average Rule of Law Index score for 2005-2015, z-standardised with a mean of zero and standard deviation of 1.

Note 2: n-sizes for all models. Pupil level (n=222,547), average pupils per country (n=4,280), countries (n=52).

Note 3: All models include t-1 dummies for year to account for time-trends (not tabled).

**OS Table 6: Multilevel models for intermittent to frequent fighting (2-3+ times) and sex in 63 countries**

|  | **Model 1**  **Empty model** | | | **Model 2**  **Random intercept** | | | **Model 3**  **Random slope** | | |
| --- | --- | --- | --- | --- | --- | --- | --- | --- | --- |
|  | **OR** | **95% CI** | **p** | **OR** | **95% CI** | **p** | **OR** | **95% CI** | **p** |
| *Intercept* | 0.52 | (0.43, 0.62) | 0.000 | 0.32 | (0.26,0.38) | 0.000 | 0.30 | (0.24,0.37) | 0.000 |
| Sex |  |  |  | 2.68 | (2.62,2.74) | 0.000 | 2.62 | (2.33,2.96) | 0.000 |
| *Random effects* |  |  |  |  |  |  |  |  |  |
| Intercept variance | 0.39 | (0.27,0.56) |  | 0.40 | (0.28,0.58) |  | 0.56 | (0.39,0.81) |  |
| L1 coeff. variance | |  |  |  |  |  | 0.23 | (0.16,0.33) |  |
| Cov(coefficient) |  |  |  |  |  |  | -0.21 | (-0.31,-0.10) |  |
| ICC | 10.55% | (7.61, 14.43) |  | 10.90% | (7.88, 14.89) |  | 14.56% | (10.61, 19.68) |  |
| Log likelihood |  | -120558.27 |  |  | -116123.04 |  |  | -115340.01 |  |
| Likelihood ratio test |  |  |  | M2 vs M1: | | | M3 vs M2: | | |
|  |  |  |  | *χ^2^* (1 df) 8870.47; p<.001 | | | *χ^2^ (*2 df) 1566.06; p<.001 | | |

Note 1: All models include t-1 dummies for year to account for time-trends (not tabled).

Note 2: n-sizes for all models. Pupil level (n=247,909), average pupils per country (n=3,935), countries (n=63).

**OS Table 7: Multilevel logistic regression models for prevalence of intermittent to frequent fighting (2-3+ times), average gender inequality index and sex**

|  | **Model 1 GII**  **Empty model** | | | **Model 2 GII**  **Random intercept** | | | **Model 3 GII**  **Random intercept** | | | **Model 4 GII**  **Random slope** | | | **Model 5 GII**  **Cross-level interaction** | | |
| --- | --- | --- | --- | --- | --- | --- | --- | --- | --- | --- | --- | --- | --- | --- | --- |
|  | **OR** | **95% CI** | **p** | **OR** | **95% CI** | **p** | **OR** | **95% CI** | **P** | **OR** | **95% CI** | **P** | **OR** | **95% CI** | **P** |
| *Intercept* | 0.45 | (0.37, 0.55) | 0.000 | 0.43 | (0.35, 0.52) | 0.000 | 0.26 | (0.22, 0.32) | 0.000 | 0.25 | (0.20, 0.31) | 0.000 | 0.23 | (0.19, 0.29) | 0.000 |
| Sex |  |  |  |  |  |  | 2.66 | (2.60,2.72) | 0.000 | 2.73 | (2.38,3.13) | 0.000 | 2.97 | (2.60,3.38) | 0.000 |
| *Country level: Av. GII* |  |  |  | 1.15 | (0.99,1.32) | 0.062 | 1.14 | (0.98,1.32) | 0.079 | 1.12 | (0.95,1.32) | 0.162 | 1.3 | (1.10,1.53) | 0.002 |
| *Cross-level interaction* |  |  |  |  |  |  |  |  |  |  |  |  | 0.81 | (0.72,0.90) | 0.000 |
| *Random effects* |  |  |  |  |  |  |  |  |  |  |  |  |  |  |  |
| Intercept variance | 0.32 | (0.23,0.51) |  | 0.32 | (0.21,0.48) |  | 0.33 | (0.22,0.49) |  | 0.43 | (0.28,0.66) |  | 0.4 | (0.27,0.61) |  |
| L1 coeff. variance |  |  |  |  |  |  |  |  |  | 0.24 | (0.16,0.37) |  | 0.19 | (0.13,0.29) |  |
| Cov(coefficient) |  |  |  |  |  |  |  |  |  | -0.16 | (-0.27,-0.05) |  | -0.12 | (-0.21,-0.04) |  |
| ICC | 9.42% | (6.51, 13.44) |  | 8.86% | (6.11, 12.69) |  | 9.05% | (6.24, 12.94) |  | 11.51% | (7.84, 16.61) |  | 10.95% | (7.57, 15.57) |  |
| Log likelihood |  | -106324.22 |  |  | -106322.54 |  |  | -102501.66 |  |  | -101796.72 |  |  | -101790.53 |  |
| Likelihood ratio test |  |  |  | M2 vs M1: | | | M3 vs M2: | | | M4 vs M3: | | | M5 vs M4: | | |
|  |  |  |  | *χ^2^* (1 df): 3.36; p=0.067 | | | *χ^2^* (1 df): 7641.76; p<.001 | | | *χ^2^* (2 df): 1409.88; p<.001 | | | *χ^2^* (1 df): 12.37; p<.001 | | |

Note 1: GII is the average Gender Inequality Index for 2005-2015, z-standardized with a mean of zero and standard deviation of 1.

Note 2: n-sizes for all models. Pupil level (n=222,547), average pupils per country (n=4,280), countries (n=52).

Note 3: All models include t-1 dummies for year to account for time-trends (not tabled).

**OS Table 8: Multilevel logistic regression models for intermittent to frequent fighting (2-3+ times), average rule of law index and sex**

|  | **Model 1 Rule of Law**  **Random intercept** | | | **Model 2 Rule of Law**  **Random intercept** | | | **Model 3 Rule of Law**  **Random slope** | | | **Model 4 Rule of Law**  **Cross-level interaction** | | |
| --- | --- | --- | --- | --- | --- | --- | --- | --- | --- | --- | --- | --- |
|  | **OR** | **95% CI** | **p** | **OR** | **95% CI** | **P** | **OR** | **95% CI** | **P** | **OR** | **95% CI** | **P** |
| *Intercept* | 0.49 | (0.40, 0.60) | 0.000 | 0.30 | (0.25, 0.37) | 0.000 | 0.28 | (0.22, 0.35) | 0.000 | 0.28 | (0.23, 0.36) | 0.000 |
| Sex |  |  |  | 2.66 | (2.60,2.72) | 0.000 | 2.73 | (2.38,3.13) | 0.000 | 2.69 | (2.32,3.12) | 0.000 |
| *Country level: Av. Rule of Law* | 1.24 | (1.05,1.48) | 0.014 | 1.25 | (1.05,1.49) | 0.011 | 1.24 | (1.04,1.47) | 0.015 | 1.28 | (1.04,1.57) | 0.020 |
| *Cross-level interaction* | |  |  |  |  |  |  |  |  | 0.96 | (0.82,1.12) | 0.590 |
| *Random effects* | |  |  |  |  |  |  |  |  |  |  |  |
| Intercept variance | 0.31 | (0.21,0.46) |  | 0.31 | (0.21,0.46) |  | 0.44 | (0.29,0.66) |  | 0.44 | (0.29,0.65) |  |
| L1 coeff. variance | |  |  |  |  |  | 0.24 | (0.16,0.37) |  | 0.24 | (0.16,0.36) |  |
| Cov(coefficient) | |  |  |  |  |  | -0.18 | (-0.29,-0.08) |  | -0.18 | (-0.29,-0.08) |  |
| ICC | 8.52% | (5.87, 12.20) |  | 8.58% | (5.92, 12.29) |  | 11.75% | (8.17, 16.62) |  | 11.73% | (8.16, 16.59) |  |
| Log likelihood | | -106321.33 |  |  | -102500.09 |  |  | -101794.88 |  |  | -101794.73 |  |
| Likelihood ratio test | M1 vs. Empty model | | | M3 vs M2: | | | M4 vs M3: | | | M5 vs M4: | | |
|  | *χ^2^* (1 df): 5.79; p<.05 | | | *χ^2^* (1 df):7642.47; p<.001 | | | *χ^2^* (2 df): 1410.43; p<.001 | | | *χ^2^* (1 df): 0.29; p=.591 | | |

Note 1: Rule of Law is the average Rule of Law Index score for 2005-2015, z-standardised with a mean of zero and standard deviation of 1.

Note 2: n-sizes for all models. Pupil level (n=222,547), average pupils per country (n=4,280), countries (n=52).

Note 3: All models include t-1 dummies for year to account for time-trends (not tabled).

**OS Table 9: Multilevel logistic regression models for frequent fighting, income inequality, and sex**

|  | **Model 1 GINI** | | | **Model 2 GINI** | | | **Model 3 GINI** | | | **Model 4 GINI** | | | **Model 5 GINI** | | |
| --- | --- | --- | --- | --- | --- | --- | --- | --- | --- | --- | --- | --- | --- | --- | --- |
|  | **OR** | **95% CI** | **p** | **OR** | **95% CI** | **p** | **OR** | **95% CI** | **p** | **OR** | **95% CI** | **p** | **OR** | **95% CI** | **p** |
| *Intercept* | 0.21 | (0.16, 0.27) | 0.000 | 0.21 | (0.16, 0.27) | 0.000 | 0.13 | (0.10, 0.17) | 0.000 | 0.12 | (0.09, 0.16) | 0.000 | 0.12 | (0.09, 0.16) | 0.000 |
| Sex |  |  |  |  |  |  | 2.50 | (2.42, 2.58) | 0.000 | 2.64 | (2.28, 3.06) | 0.000 | 2.6 | (2.26, 3.01) | 0.000 |
| *Country level: GINI* | |  |  | 1.03 | (0.84, 1.27) | 0.755 | 1.04 | (0.84, 1.27) | 0.743 | 0.98 | (0.80, 1.20) | 0.850 | 1.1 | (0.86, 1.41) | 0.440 |
| *Cross-level interaction* | | |  |  |  |  |  |  |  |  |  |  | 0.89 | (0.78, 1.03) | 0.119 |
| *Random effects* | |  |  |  |  |  |  |  |  |  |  |  |  |  |  |
| Intercept variance | 0.53 | (0.35, 0.80) |  | 0.53 | (0.35, 0.80) |  | 0.53 | (0.35, 0.79) |  | 0.77 | (0.51, 1.17) |  | 0.76 | (0.50, 1.14) |  |
| L1 coeff. variance | |  |  |  |  |  |  |  |  | 0.26 | (0.17, 0.40) |  | 0.24 | (0.16, 0.38) |  |
| Cov(coefficient) | |  |  |  |  |  |  |  |  | -0.28 | (-0.44, -0.12) |  | -0.26 | (-0.41, -0.11) |  |
| ICC | 13.89% | (9.66, 19.59) |  | 13.90% | (9.67, 19.58) |  | 13.77% | (9.57, 19.40) |  | 18.99% | (13.43, 26.16) |  | 18.69% | (13.25, 25.71) |  |
| Log likelihood | | -62735.41 |  |  | -62735.37 |  |  | -61042.11 |  |  | -60716.65 |  |  | -60715.48 |  |
| Likelihood ratio test |  |  |  | M2 vs M1: | | | M3 vs M2: | | | M4 vs M3: | | | M5 vs M4: | | |
|  |  |  |  | *χ^2^* (1 df): 0.10; p=0.755 | | | *χ^2^* (1 df): 3386.52; p<.001 | | | *χ^2^* (2 df): 650.92; p<.001 | | | *χ^2^* (1 df): 2.34; p=0.126 | | |

Note 1: Income inequality is measured by the GINI Index, z-standardised with a mean of zero and standard deviation of 1.

Note 2: n-sizes for all models. Pupil level (n=215,622), average pupils per country (n=4,228), countries (n=51).

Note 3: All models include t-1 dummies for year to account for time-trends (not tabled).

**OS Table 10: Multilevel logistic regression models for any fighting (1+ times), income inequality, and sex**

|  | **Model 1 GINI** | | | **Model 2 GINI** | | | **Model 3 GINI** | | | **Model 4 GINI** | | | **Model 5 GINI** | | |
| --- | --- | --- | --- | --- | --- | --- | --- | --- | --- | --- | --- | --- | --- | --- | --- |
|  | **OR** | **95% CI** | **p** | **OR** | **95% CI** | **p** | **OR** | **95% CI** | **p** | **OR** | **95% CI** | **p** | **OR** | **95% CI** | **p** |
| *Intercept* | 0.98 | (0.82, 1.17) | 0.831 | 0.98 | (0.82, 1.18) | 0.839 | 0.64 | (0.53, 0.77) | 0.000 | 0.59 | (0.48, 0.74) | 0.000 | 0.60 | (0.49, 0.74) | 0.000 |
| Sex |  |  |  |  |  |  | 2.56 | (2.51, 2.61) | 0.000 | 2.54 | (2.22, 2.91) | 0.000 | 2.50 | (2.19, 2.85) | 0.000 |
| *Country level: GINI* | |  |  | 1.01 | (0.87, 1.18) | 0.862 | 1.02 | (0.87, 1.19) | 0.830 | 0.97 | (0.83, 1.13) | 0.683 | 1.09 | (0.91, 1.32) | 0.351 |
| *Cross-level interaction* | | |  |  |  |  |  |  |  |  |  |  | 0.87 | (0.76, 0.99) | 0.033 |
| *Random effects* | |  |  |  |  |  |  |  |  |  |  |  |  |  |  |
| Intercept variance | 0.30 | (0.20, 0.45) |  | 0.30 | (0.20, 0.45) |  | 0.31 | (0.21, 0.47) |  | 0.46 | (0.31, 0.70) |  | 0.45 | (0.30, 0.67) |  |
| L1 coeff. variance | |  |  |  |  |  |  |  |  | 0.24 | (0.16, 0.36) |  | 0.22 | (0.15, 0.33) |  |
| Cov(coefficient) | |  |  |  |  |  |  |  |  | -0.21 | (-0.32, -0.10) |  | -0.19 | (-0.30, -0.09) |  |
| ICC | 8.33% | (5.74, 11.93) |  | 8.33% | (5.74, 11.93) |  | 8.69% | (6.01, 12.42) |  | 12.37% | (8.56, 17.54) |  | 12.02% | (8.37, 16.95) |  |
| Log likelihood | | -136782.39 |  |  | -136782.38 |  |  | -131736.30 |  |  | -130768.15 |  |  | -130765.98 |  |
| Likelihood ratio test |  |  |  | M2 vs M1: | | | M3 vs M2: | | | M4 vs M3: | | | M5 vs M4: | | |
|  |  |  |  | *χ^2^* (1 df): 0.03; p=0.862 | | | *χ^2^* (1 df): 10092.15; p<.001 | | | *χ^2^* (2 df): 1936.30; p<.001 | | | *χ^2^* (1 df): 4.34; p<.05 | | |

Note 1: Income inequality is measured by the GINI Index, z-standardised with a mean of zero and standard deviation of 1.

Note 2: n-sizes for all models. Pupil level (n=215,622), average pupils per country (n=4,228), countries (n=51).

Note 3: All models include t-1 dummies for year to account for time-trends (not tabled).

**OS Table 10: Multilevel logistic regression models for any fighting (2-3+ times), income inequality, and sex**

|  | **Model 1 GINI** | | | **Model 2 GINI** | | | **Model 3 GINI** | | | **Model 4 GINI** | | | **Model 5 GINI** | | |
| --- | --- | --- | --- | --- | --- | --- | --- | --- | --- | --- | --- | --- | --- | --- | --- |
|  | **OR** | **95% CI** | **p** | **OR** | **95% CI** | **p** | **OR** | **95% CI** | **p** | **OR** | **95% CI** | **p** | **OR** | **95% CI** | **p** |
| *Intercept* | 0.48 | (0.40, 0.59) | 0.000 | 0.48 | (0.39, 0.59) | 0.000 | 0.3 | (0.25, 0.37) | 0.000 | 0.28 | (0.22, 0.35) | 0.000 | 0.28 | (0.22, 0.36) | 0.000 |
| Sex |  |  |  |  |  |  | 2.54 | (2.49, 2.60) | 0.000 | 2.58 | (2.25, 2.97) | 0.000 | 2.55 | (2.23, 2.91) | 0.000 |
| *Country level: GINI* | |  |  | 0.99 | (0.83, 1.18) | 0.928 | 0.99 | (0.83, 1.18) | 0.943 | 0.95 | (0.80, 1.12) | 0.523 | 1.07 | (0.87, 1.31) | 0.545 |
| *Cross-level interaction* | | |  |  |  |  |  |  |  |  |  |  | 0.88 | (0.77, 1.00) | 0.059 |
| *Random effects* | |  |  |  |  |  |  |  |  |  |  |  |  |  |  |
| Intercept variance | 0.37 | (0.25, 0.56) |  | 0.37 | (0.25, 0.56) |  | 0.38 | (0.25, 0.57) |  | 0.57 | (0.37, 0.85) |  | 0.55 | (0.37, 0.82) |  |
| L1 coeff. variance | |  |  |  |  |  |  |  |  | 0.24 | (0.16, 0.37) |  | 0.22 | (0.15, 0.34) |  |
| Cov(coefficient) | |  |  |  |  |  |  |  |  | -0.23 | (-0.36, -0.10) |  | -0.22 | (-0.34, -0.10) |  |
| ICC | 10.19% | (7.05, 14.51) |  | 10.18% | (7.04, 14.50) |  | 10.35% | (7.16, 14.73) |  | 14.66% | (10.23, 20.56) |  | 14.33% | (10.05, 20.04) |  |
| Log likelihood | | -104167.63 |  |  | -104167.63 |  |  | -100729.57 |  |  | -100069.98 |  |  | -100068.26 |  |
| Likelihood ratio test |  |  |  | M2 vs M1: | | | M3 vs M2: | | | M4 vs M3: | | | M5 vs M4: | | |
|  |  |  |  | *χ^2^* (1 df): 0.01; p=0.928 | | | *χ^2^* (1 df): 6876.12; p<.001 | | | *χ^2^* (2 df): 1319.18; p<.001 | | | *χ^2^* (1 df): 3.44; p=0.064 | | |

Note 1: Income inequality is measured by the GINI Index, z-standardised with a mean of zero and standard deviation of 1.

Note 2: n-sizes for all models. Pupil level (n=215,622), average pupils per country (n=4,228), countries (n=51).

Note 3: All models include t-1 dummies for year to account for time-trends (not tabled).

OS Figure 1: Bivariate distribution of random slope and intercept (n=63 countries)

OS Figure 2: Estimated marginal effect of sex on any fighting (1+ times) by country-level income inequality (n=51 countries)
